# A functional magnetic resonance imaging investigation of visual hallucinations in the human striate cortex

**DOI:** 10.1186/s12993-016-0115-y

**Published:** 2016-11-29

**Authors:** Hina Abid, Fayyaz Ahmad, Soo Y. Lee, Hyun W. Park, Dongmi Im, Iftikhar Ahmad, Safee U. Chaudhary

**Affiliations:** 1 Quaid-e-Azam University, Islamabad, Pakistan; 2 University of Gujrat, Gujrat, Pakistan; 3 Korea Advanced Institute of Science and Technology, Daejeon, South Korea; 4 Lahore University of Managment Sciences, Lahore, Pakistan

**Keywords:** Functional magnetic resonance imaging (fMRI), Visual hallucinations, Visual cortex, Brodmann area, K-means clustering, Logistic regression

## Abstract

**Purpose:**

Human beings frequently experience fear, phobia, migraine and hallucinations, however, the cerebral mechanisms underpinning these conditions remain poorly understood. Towards this goal, in this work, we aim to correlate the human ocular perceptions with visual hallucinations, and map them to their cerebral origins.

**Methods:**

An fMRI study was performed to examine the visual cortical areas including the striate, parastriate and peristriate cortex in the occipital lobe of the human brain. 24 healthy subjects were enrolled and four visual patterns including hallucination circle (HCC), hallucination fan (HCF), retinotopy circle (RTC) and retinotopy cross (RTX) were used towards registering their impact in the aforementioned visual related areas. One-way analysis of variance was used to evaluate the significance of difference between induced activations. Multinomial regression and and K-means were used to cluster activation patterns in visual areas of the brain.

**Results:**

Significant activations were observed in the visual cortex as a result of stimulus presentation. The responses induced by visual stimuli were resolved to Brodmann areas 17, 18 and 19. Activation data clustered into independent and mutually exclusive clusters with HCC registering higher activations as compared to HCF, RTC and RTX.

**Conclusions:**

We conclude that small circular objects, in rotation, tend to leave greater hallucinating impressions in the visual region. The similarity between observed activation patterns and those reported in conditions such as epilepsy and visual hallucinations can help elucidate the cortical mechanisms underlying these conditions.

*Trial Registration* 1121_GWJUNG

## Background

Cerebrum forms the largest part of human brain. It comprises of an outer layer called the cerebral cortex which can be further divided into four lobes namely frontal, parietal, occipital and temporal lobe [1]. Cytoarchitectonically, the cerebral cortex has been classified into 52 cortical Brodmann areas (BA) of which the occipital lobe containing the visual cortex has BAs 17, 18 and 19 [2]. Visual tasks processing related area ‘V1’ is located in BA 17 (striate cortex) while ‘V2–V6’ are located in BA 18 (parastriate cortex) and 19 (peristriate cortex). The ventral stream (‘what pathway’) initiates with V1, passes through V2 and V4, and leads into the inferior temporal cortex (IT cortex). The dorsal stream (‘where pathway’) starts at V1 and proceeds to V2, V6 and V5.

Upon absorption of light rays emitted by an object, the photoreceptors in the retina send a signal through the optic nerve via the optic chiasma into the intra laminar nucleus of the thalamus. The signal then enters V1 where the striate cortex processes the stimulus in the visual cortex of the brain in tandem with extrastriate cortex. As a result, increased blood-oxygen-level dependent (BOLD) activations can be measured in the corresponding areas of the brain. The intensity of each activation depends on the physical form of the object presented to the subject [3, 4]. Causal networking among different brain localities has been determined by Ahmed et al. [5]. The neural activations are adjudged according to the object presented and their magnitude depends on the type of the stimulus [6, 7]. Functional magnetic resonance imaging (fMRI) enables us to capture such activations in the brain, during the working phase, for onwards analysis [8, 9]. Tootell et al. [10] have reported that middle temporal (MT) region of the brain responds selectively to moving (translating or rotating) and stationary visual stimuli. Howard et al. [11], have demonstrated the effectiveness of fMRI scanners in capturing visual hallucinations in the visual cortex of patients suffering from Charles Bonnet Syndrome (CBS). It is important to note here that sometimes non-existent objects are reportedly visualized by subjects which are primarily due to residual information present in the visual cortex from past experiences [12]. Research into such observations has shown activations in V1 region of the brain suggesting that the impact of hallucinatory patterns constitutes similar cortical characteristics as that of ordinary vision. Hallucinations have also been attributed to the specific anatomical structure of the brain as proposed in the neuroanatomical model [13].

Visual hallucinations are therefore those sensory perceptions that are felt in the absence of any physical stimulus. Visual hallucinations may instigate with auras preceding petit mal epilepsy [14], fortification patterns of migraine headaches [15], drug induced hallucinations [16]. The false images comprising a visual hallucination may have either formed or unformed appearances. A person suffering from hallucinations may report seeing huge shadows, flashes of light, haphazard or outlined patterns, and may even catch a glimpse of a departed loved one. The brain may also present an oversized projection of an article which in reality may just be a minute entity. Here, it must be noted that continual experience of visual hallucinations can translate into serious human ailments such as migraine pain and epilepsy [17].

Empirically, visual hallucinations can be investigated by exposing subjects to visual stimuli consisting of the hallucinogenic patterns which may activate visual cortex of the brain [18]. Bressloff et al. [19], presented four types of images including spirals, cobwebs, tunnels and lattices and identified them as the origin of hallucinations. Stripes, spirals, rings and collective burst type patterns excite the neurons in visual cortex when exposed to the human eye [20]. A mathematical theory of such geometric type patterns, giving rise to visual hallucination, was proposed by Ermentrout et al. [21]. Vincent et al. [22] used flickering checkerboard as stimuli and measured hallucinogenic activations in brain. However, a mechanistic understanding of these induced hallucinations in the visual cortex remains elusive till date. Specifically, evaluation of hallucinogenic impacts (such as cortical magnification and retinotopy) of moving and stationary visual stimuli on BAs 17, 18 and 19 and statistical evaluation of incumbent BOLD signal data remains to be investigated.

In this study, we aim to determine if hallucinations can be induced by visual stimuli designed using cues provided by previous studies; evaluate significance and classify the hallucinogenic impacts of these visual stimuli on the visual cortical areas. Towards this goal, we induced visual hallucinations in healthy individuals by presenting them with four visual stimuli namely retinotopy cross (RTX), hallucination fan (HCF), retinotopy circle (RTC) and hallucination circle (HCC). HCF and HCC were in rotary motion about their axis while RTC and RTX were stationary. The activations registered in the visual cortex were measured using an fMRI scanner and contribution of each visual stimulus in activating the visual cortex was found to be significant corresponding to p ≤ 0.05 (FWE- correction). Finally, the mixed activation data was clustered using K-means whereby it resolved into respective BAs (17, 18 and 19). Our results show that visual cortex exhibited significant activations upon presentation of each visual stimulus with highest activations observed for HCC proceeded by RTC, HCF and RTX in order. Application of least square difference (LSD) test on the activation data identified BA 17 to be the most significant contributor to induced visual hallucinations followed by BAs 18 and 19. Moreover, the mixed activation data obtained from presentation of four stimuli was separable into individual clusters with HCC and RTX significantly activating BAs 17–19 while RTC managing activations in BA 17 only.

Taken together, we conclude that small circular objects in rotation induce greater activations in the visual cortex of the brain. These activation patterns observed are similar to those reported in migraine pain and epilepsy. Hence, the proposed experimental and data analysis methodology can assist in enhancing the understanding of visual hallucinations in disease states by an accurate cortical mapping of the brain.

## Methods

### fMRI Experimental design and data acquisition

The fMRI scanning procedure was conducted at Korea Advanced Institute of Science and Technology (KAIST), South Korea. 24 healthy subjects (15 males, 9 females, mean age 21, SD 0.8), with normal color vision, were enrolled and scanned in the study. Each subject was exposed to a procedure comprising alternating rest and task conditions while being examined by an fMRI scanner. The data obtained was preprocessed to identify and filter out datasets which contained head movement induced motion blur, background noise or low quality measurements. 4 subjects which produced the highest quality datasets were selected for onward methodological study and analysis.

Every experimental session lasted for 160 s and consisted of 8 blocks with each rest block leading a stimulus block. The duration of each rest and stimulus block was for 24 and 16 s, respectively. Within a single scanning session, 80 volume scans (32 with stimuli and 48 at rest) were obtained at intervals of 2 s. We applied cluster analysis on the resulting 80 data points corresponding to average activations in BA17, 18 and 19 voxels for the classification amongst HCC, HCF, RTC and RTX.

**Fig. 1:**
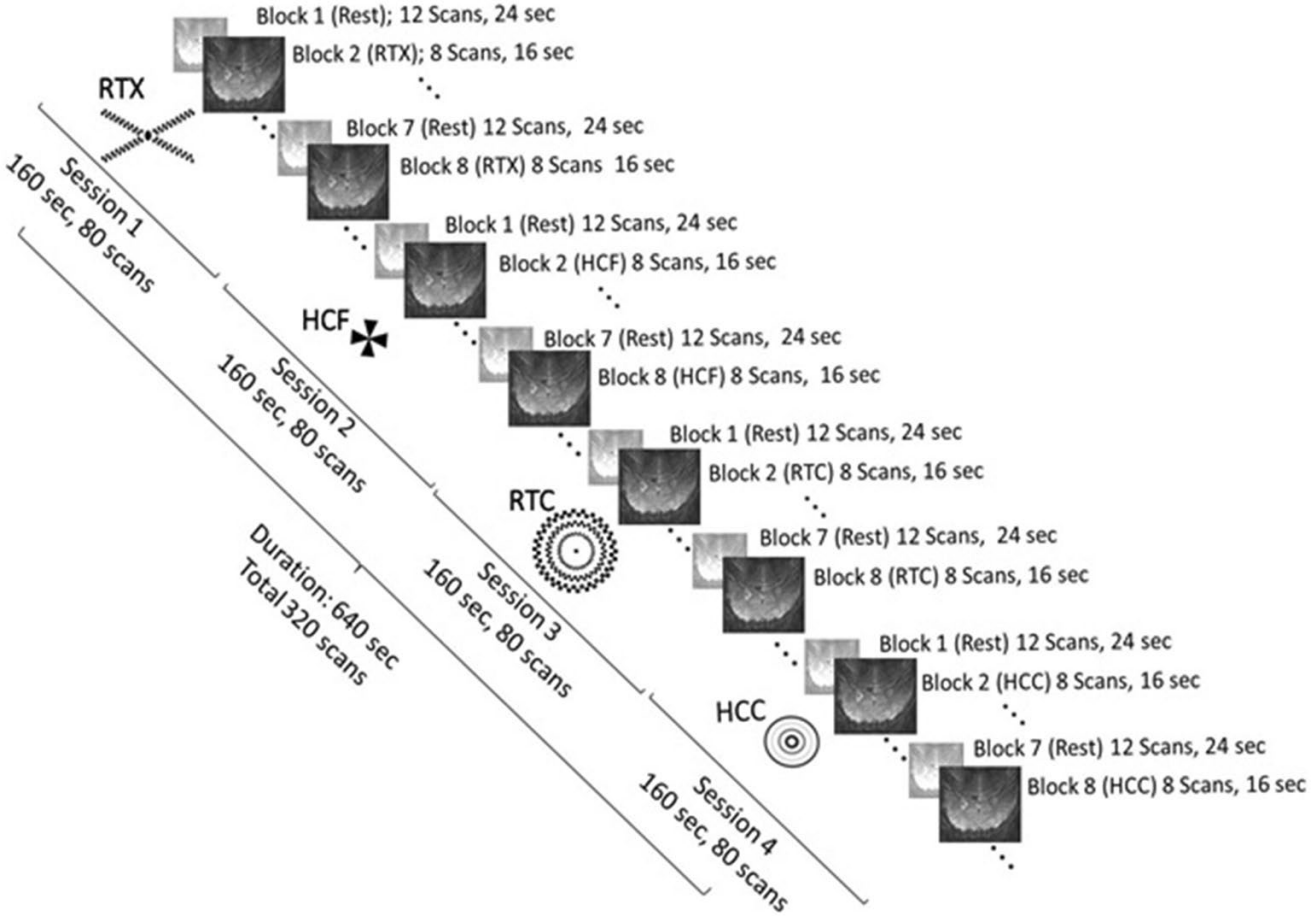
Block representation of rest and stimulus presentation phases during fMRI scanning of a participant’s brain, for *HCC*, *HCF*, *RTC* and *RTX*, in 4 sessions. Each session remained for 160 s and comprised of 8 blocks with alternative rest and stimulus phases. 80 scans were taken in each session

Four visual stimuli of different shapes and sizes were designed towards evaluating their potential hallucinogenic impact on the visual cortex. HCC comprised of concentric circles with varying diameters and colors (shades of grey). These circles were then set into synchronous rotation about the center point. RTC contained three static concentric circles with checkered boundaries and their center point indicated by a black spot. HCF pattern was a four-winged fan rotating about its center while the RTX was a stationary cross drawn using checkered lines (Fig. 1). Of the four sessions, each session was confined to a single visual stimulus. The experimental sessions were designed such that RTX was presented first followed by HCF, RTC and HCC respectively. A scan was acquired every 2 s while the stimulus was being shown. This pattern was repeated for the remaining three sessions as well. During each scan, the subjects were directed to continually focus on the presented stimulus and encouraged to keep their minds relaxed during the rest phase. To ensure high quality data from scanning procedure, their heads were placed in a brace and adjusted before a scan was performed.

### Image acquisition

The images were acquired using a 3 Tesla (FORTE, Oxford magnet, Varian Console, built up by ISOL) instrument, with a quadrature head coil to get an anatomic scan and a surface coil to obtain the functional scan. High-resolution anatomic images (structural resolution 1.25 mm isotropic voxels) were acquired using an MPRAGE sequence (echo time TE = 3.7 ms, TR = 8.1 ms, flip angle = 8°, FOV = 256 × 256 mm) and functional data were acquired using echo planar imaging (EPI, TE = 37 ms, phase encoding = top to bottom, flip angle = 70°, TR = 2000 ms, matrix = 128 × 128 mm, slices = 15, voxels = 3 mm × 3 mm, no gap) as shown Fig. 1.

### Image analysis

Data was analyzed using Statistical Parametric Mapping software (SPM8b; Wellcome Department of Cognitive Neurology, University College London, London, UK). Images realignment was performed to correct for the artifacts due to minor head vibrations and normalized to a standard Montreal Neurological Institute (MNI) template. Smoothing was done by 4-mm full width at half maximum smoothing to average the data with the neighboring data points. Images were analyzed using contrast vector C = [1 −1] corresponding to p < 0.05 [Family Wise Error (FWE) correction] (Fig. 3).

### fMRI images configuration

Grey-scale fMRI images were used for onward investigations, with the darker regions having a higher pixel value while the lighter regions approaching to a zero on the pixel scale. A total of 80 scans were taken for each session so as to ascertain accuracy in the ensuing statistical analysis of these results. All volume scans were cut down into 15 slices. The dimension of a single volume was 128 × 128 × 15. The total number of voxels in a volume counted to 245,760. Each voxel in the study had a uniform size of 3 × 3 × 3 mm.

### Statistical methods and techniques for fMRI data analysis

To test the variation and significance of data obtained after presentation of visual stimuli, analysis of variance (ANOVA) test [23] was employed. Upon ascertaining significance of impact on visual cortex, LSD [24] was applied to determine the individual contribution of each stimulus on the visual cortex. For classifying the mixed cortical activations into clusters, K-means clustering [25] was applied to the activation data. To compute the probabilistic relationship between each visual stimuli and BAs 17, 18 and 19, we used multinomial logistic regression (MLR) [26].

## Results

### Analysis of fMRI data obtained from presentation of four visual stimuli

**Fig. 2:**
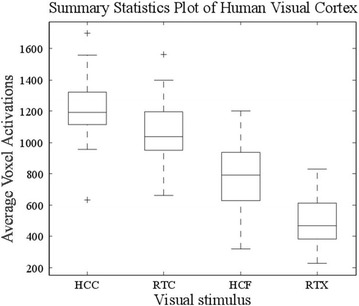
Summary statistics plot of human visual cortex for four visual stimulus. The visual stimulus types are taken on *x-axis* and the average voxel activations are on *y-axis*. The *boxplot* displays the average voxel activations in the visual cortex of a single participant for each visual stimulus

**Fig. 3:**
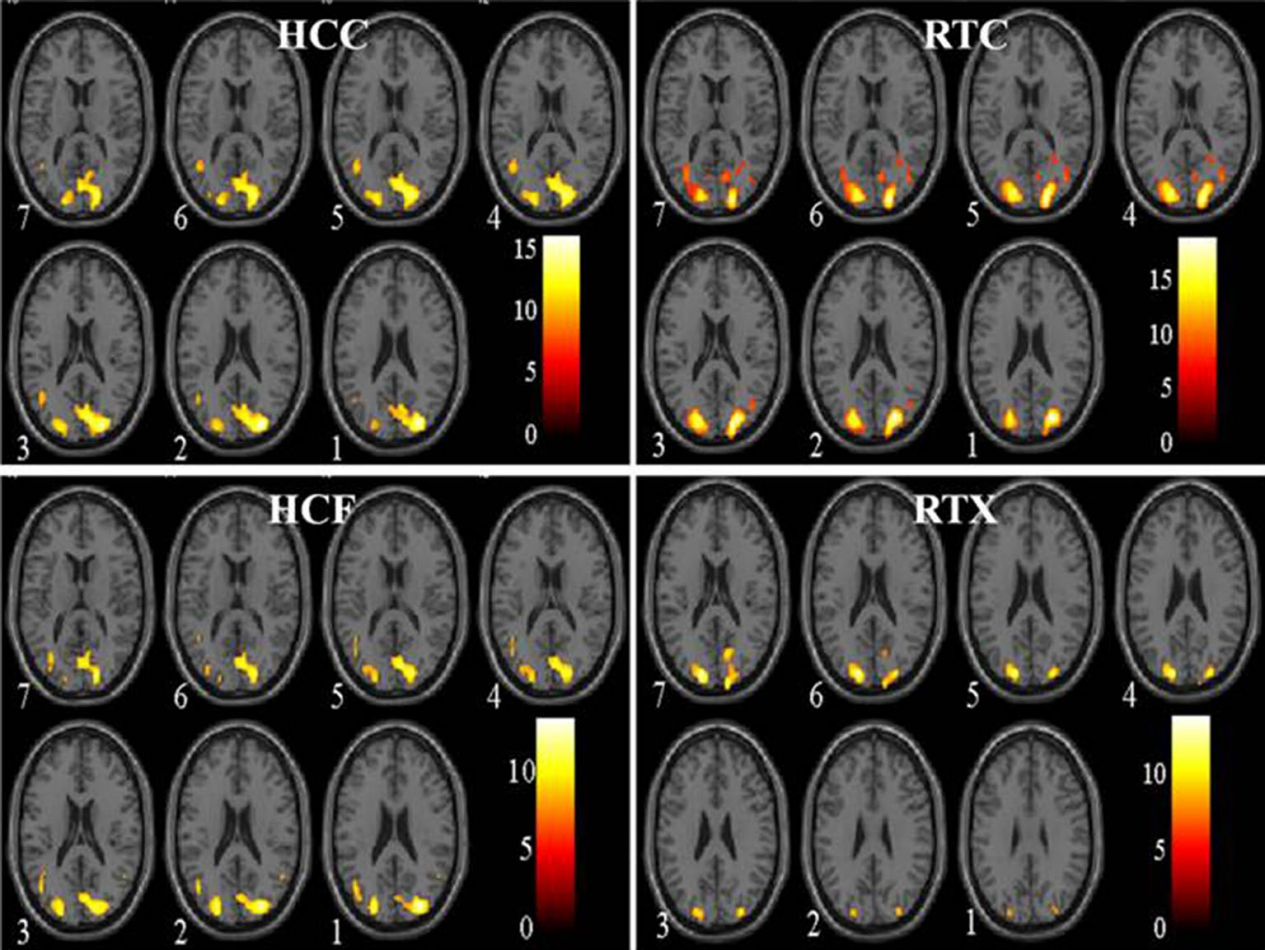
Comparison of activated areas in the visual cortex of a subject. The figure shows a 2 × 2 display of statistical parameter maps (SPM) after presenting *HCC*, *RTC*, *HCF* and *RTX*. The significant activations are measured using t-scale at p < 0.05 (corrected)

Upon presentation of visual stimuli, BOLD signals were measured in the visual cortex. The axial slicing view of fMRI scans was observed and activations were registered only in the middle axial slices (MNI coordinates and cluster size in Table 1). The activation data obtained was continuous time-series fMRI data. These activations were evaluated using *t* test (p < 0.05 FEW-corrected), for each scan (task vs. rest state), and exhibited varying levels of activation in each case (Fig. 2). Highest activations were determined by comparing the averages of voxel activations induced by the four stimuli, in a participant’s visual cortex, using general linear model (GLM) analysis. The distribution of voxel activations for a single participant, for each stimulus, is shown in Fig. 3. Highest activations were observed when HCC came into sight (average: 1190) followed by RTC (average: 1050), HCF (average: 796) and RTX (average: 475), in order as shown in box plot. The results also showed that HCF elicited the most variable response followed by RTC, HCC and RTX, in descending order. The participants reported magnified visualizations in cases of HCC and RTC stimuli. The four conditions can, therefore, be discriminated from each other based on the fMRI responses elicited from 3 visual areas. Importantly, the hallucinatory stimuli can excite more neurons than normal retinotopic stimuli.

**Table 1:** MNI coordinates of peak voxels within each cluster and T statistics from BA17 (Threshold = p < 0.05)

ROI	Stimulus	x	y	z	T value	Cluster size
R V1	HCC	11	−78	7	15.41	3941
L V1	HCC	−11	−81	8	15.62	1486
R V1	HCF	17	−83	11	17.47	4698
L V1	HCF	−7	−84	13	16.07	2254
R V1	RTC	14	−88	8	16.07	2251
L V1	RTC	−8	−75	15	17.47	4700
R V1	RTX	19	−74	12	15.62	893
L V1	RTX	−3	−76	6	22.50	1342

### Significance analysis of cortical activation data

To ascertain the significance of the stimulus-induced activations, ANOVA testing was employed. Our results (Table 2) show that all stimuli had exerted a significant impact on the visual cortex (p < 0.05). Furthermore, a pairwise comparison (using LSD test) was performed between the activation data of each stimulus towards computing the contribution of stimuli activating the visual cortex. HCC’s pair-wise contribution (RTC = 79.9757825, HCF = 40.0077425, RTX = 17.5432856) was found to be the largest, followed by RTC (HCC = 79.9757825, HCF = 39.9680400, RTX = 62.4324969), HCF (HCC = 40.0077425, RTC = 39.9680400, RTX = 22.4644569) and RTX (HCC = 17.5432856, RTC = 62.4324969, HCF = 22.4644569), in terms of magnitude (Table 3).

**Table 2:** One way analysis of variance

Variation source	Sum of squares	df	Mean square	F	Sig.
Between groups	286,371.146	3	95,457.049	1056.665	0.000
Within groups	28,637.164	317	90.338		
Total	315,008.310	320			

The mean difference is significant at the 0.05 level

**Table 3:** LSD test for significance

(I) Factors	(J) Factor	Mean difer (I – J)	Std. error	Sig.
HCC	RTC	−79.9757825*	1.5028145	.000
	HCF	−40.0077425*	1.5028145	.000
	RTX	−17.5432856*	1.4981690	.000
RTC	HCC	79.9757825*	1.5028145	.000
	HCF	39.9680400*	1.5028145	.000
	RTX	62.4324969*	1.4981690	.000
HCF	HCC	40.0077425*	1.5028145	.000
	RTC	−39.9680400*	1.5028145	.000
	RTX	22.4644569*	1.4981690	.000
RTX	HCC	17.5432856*	1.4981690	.000
	RTC	62.4324969*	1.4981690	.000
	HCF	−22.4644569*	1.4981690	.000

* The mean difference is significant at the 0.05 level

### Classification of cortical activations by visual stimuli

**Fig. 4:**
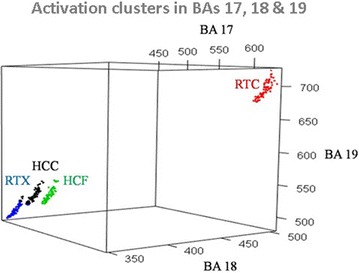
Clustering of voxel activation information for *HCC*, *HCF*, *RTC* and *RTX*, in *BA 17*, *18* and *19*. K-means clustering is used for cluster formation. The *x*, *y* and *z*—axes are labeled as the average voxel activations in *BA 17*, *18* and *19* respectively

**Fig. 5:**
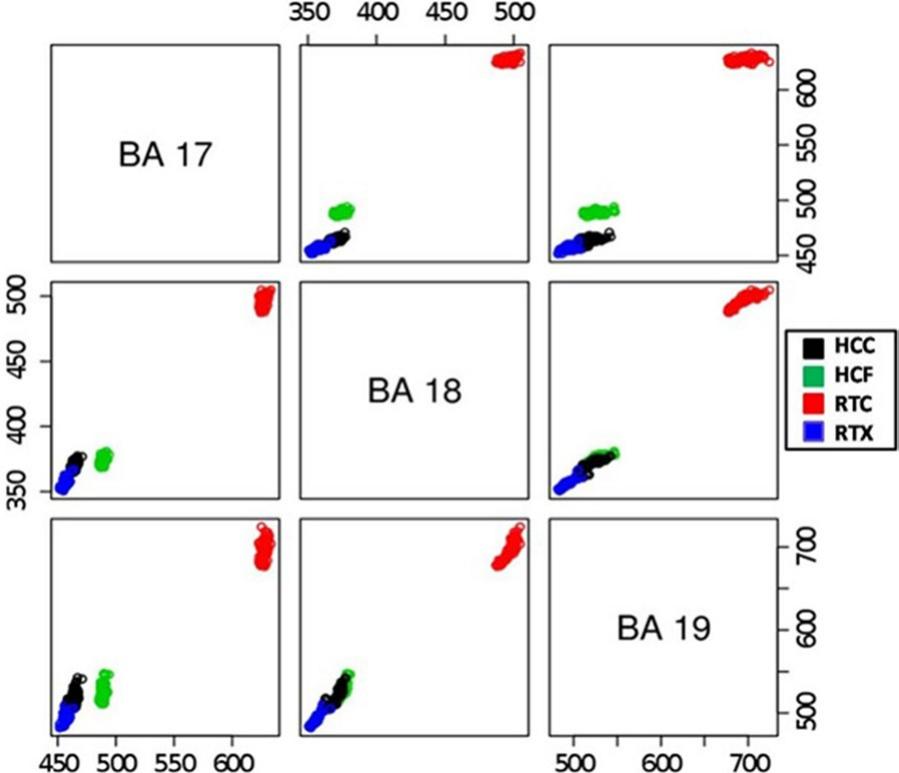
Scatter matrix plot of activated information corresponding to visual cortical areas. The *plot* is a 3 × 3 display with *BA 17*, *18* and *19* shown in *rows* (*columns*) 1, 2 and 3 respectively. The off diagonal cells represents the pairwise correlation of clustered points in BA’s. The cells in lower triangle below the diagonal are the mirror images of the cells in the upper triangle above the diagonal of the *matrix plot*

To classify the cortical activation data generated by the presented visual stimuli, K-means clustering was employed. The data got separated into clusters 1 through 4 (Fig. 4). Activations generated by HCC and RTX clustered into clusters 1 and 4, respectively. However, there was a slight mixing between these clusters (Cluster 1; HCC = 76, RTX = 6, Cluster 4; HCC = 4, RTX = 74). Data corresponding to RTC and HCF stimuli clustered perfectly into clusters 2 and 3, respectively. The within sum of square (SSE) was used to measure the cluster cohesion and it was found to be the highest (12436.723) for cluster 2 (Table 4). Qualitative analysis of clustering results was performed by computing variations within and in between clusters (total sum of square), overall cluster cohesion (total within sum of squares) and cluster separation (between sum of square) (results shown in Table 5). Classification of the voxel activations data for HCC, HCF, RTC and RTX into individual clusters determined the correlation in the three BA’s. The impact of HCC on BAs (17, 18 and 19) was found out to be 460, 370 and 530, respectively. For RTC it was 640, 500 and 710; for HCF the impact was 490, 370 and 540 while for RTX it was 460, 360 and, 500. The pair-wise activated voxels with respect to each BA (17, 18 and 19) have been plotted using a scatter matrix plot (Fig. 5; Table 6).

**Table 4:** Size of clusters, cluster means and within sum of squares

No. of cluster	Cluster size	Cluster means			Within sum of squares
		BA 17	BA 18	BA 19	
1	82	464.2945	370.8899	519.8890	7605.788
2	80	627.4581	496.4402	694.6261	12,436.723
3	80	488.8951	374.0905	524.2706	7108.980
4	78	457.0710	357.5852	495.6686	5530.045

**Table 5:** Sum of squares

Total SS	Within SS	Between SS
4,575,623	32,681.54	4,542,941

**Table 6:** Allocation of points corresponding to each cluster

	Cluster 1	Cluster 2	Cluster 3	Cluster 4
HCC	76	0	0	4
HCF	0	80	0	0
RTC	0	0	80	0
RTX	6	0	0	74

### Probabilistic relationship of visual stimulus with BA 17, 18 and 19

To determine the probabilistic relationship between the each stimulus (HCC, RTC, HCF and RTC) and every Brodmann area (BA 17, 18 and 19), multinomial logistic regression (MLR) was employed. Inferior temporal gyrus (BA 20); a visual cortical area lying in the temporal lobe of the brain was used as a reference base category. Category 1 was reserved for BA 20 while BAs 17, 18 and 19 were encoded into categories 2, 3 and 4, respectively. MLR coefficient of HCC was found to be the highest for BA 17 (45.44663) and lowest for BA 19 (22.30781) suggesting that striate cortex was highly activated by HCC (Table 7). For RTC, HCF and RTX, their respective contribution in activating BA 17 was found to be 11.378715, 2.51319718 and 36.24384. For the activations in BA 19, RTC, HCF and RTX contributed 2.449028, 0.03642327 and 20.10646, respectively.

**Table 7:** Coefficients of multinomial logistic regression model

Y	Intercept	pHCC	pRTC	pHCF	pRTX
2^a^	−0.3372760 (0.4586571)^b^	−45.44663 (34.74687)	11.378715 (9.509267)	−2.51319718 (1.4800913)	36.24384 (33.28803)
3	−0.5260880 (0.4831243)	33.22444 (23.03927)	−4.316287 (8.545127)	0.2773366 (0.8823183)	−29.71157 (21.40103)
4	−0.2112646 (0.4322987)	22.30781 (22.33585)	−2.449028 (8.040815)	0.03642327 (0.8376842)	−20.10646 20.87877

^a^Categories 2, 3 and 4 represents BA 17, 18 and 19 respectively

^b^Standard errors

## Discussion

This study aims to elucidate the cortical mechanisms underpinning visual hallucinations in the human brain thereby building an improved understanding of the condition. Towards this goal, experiments were designed around four visual stimuli (HCC, RTC, HCF and RTX), having different shapes and sizes with varying movements. These stimuli were presented to the participants and their fMRI scans were obtained. The resulting data was analyzed towards determining the impact of each visual stimulus on the visual cortex (i.e. BAs 17, 18 and 19). Significant activations were observed in the visual cortex upon presentation of each stimulus type, however, the magnitude of induced activations was observed to be different. For each participant, HCC induced the highest BOLD signal in the visual cortex followed by RTC, HCF and RTX during 80 scans. The results indicated that smaller objects having circular appearances in rotation create larger impacts in the visual cortex as compared to static non-circular objects. Furthermore, while visualizing HCC and RTC, the participants reported visual perceptions of enlarged visual stimuli which were in fact artificially induced hallucinations.

Having induced hallucinating impacts in the visual cortex of the participants, we set out to analyze the continuous time-series fMRI data using specific statistical techniques. We investigated the patterns of activations by measuring impact of each visual stimulus on each Brodmann area. Clustering of the mixed task and rest state data helped us determine the activation correlation in the three BA’s for hallucinating (HCC, RTC) and non-hallucinating (HCF, RTX) stimuli. The impact of HCC on BAs (17, 18 and 19) was found out to be 460, 370 and 530, respectively. For RTC it was 640, 500 and 710; for HCF the impact was 490, 370 and 540 while for RTX it was 460, 360 and, 500. These results, specifically for HCC and RTC, were interpreted to constitute a Brodmann area footprint as the requisite setting for experiencing visual hallucinations.

Similar studies conducted earlier have employed cobwebs, funnel, spirals and concentric circles towards inducing hallucinations [21]. In our experiments, we have designed and employed four unique visual stimuli comprising of circles (HCC, RTC) and crosses (RTX, HCF) and elicited their impact on visual cortex. The stimuli HCC and HCF were also set into motion while RTC and RTX were kept static. It might be of interest to evaluate the cortical impact of an expanded set of visual stimuli, with a broader range of optical properties. Stimuli design changes may include different shapes, sizes and, rates of rotation.

Furthermore, in case of diseases such as epilepsy and migraine, research has reported similar hallucinating impacts in the visual cortex of patients [27]. Patients are known to experience auras such as flickering, zig-zag lines, disks and balls of light [28]. These auras may be enlarged or diminished in size, stationary or moving and single or multiple. The magnified shapes that are seen by patients suffering from such pathologies are comparable to the hallucinating magnification reported by participants in our study [29]. Hence, the statistical evaluation methodology described in this work can specifically assist in eliciting the cortical mechanisms giving rise to enlargement of objects in epilepsy [27]. The study can be extended further by replacing the uncolored stimuli with colored ones, in varying shapes. This can help in benchmarking the hallucinating sensations induced in the subjects against those in the patients. Moreover, auditory, olfactory and tactile hallucination studies can also be carried out and their impacts can be measured in the respective BA’s. Furthermore, our study can also be extended by employing more powerful data analysis tools such as structural equation modelling (SEM) and Bayesian techniques towards investigating the interplay between BAs during induced hallucinations.

Taken together, the proposed methodology can be employed in investigating the impact of a variety of stimuli on the visual cortex. Alongside, the findings from this study can assist in screening as well as prognosis of epileptic and migraine patients presenting specific hallucinating patterns in fMRI analysis.

## Appendix: MATLAB program for extracting ROIs data

clear all;

BrodmannAtlas = spm_read_vols(spm_vol(`xxbrodmann.img’));

BA 17 = ismember(BrodmannAtlas,17);

V = spm_vol(`xxbrodmann.img’);

V.fname=`BA17.nii’;

V.private.dat.fname = V.fname;

spm_write_vol(V,BA 17);

BA 17;

Temp_array = [];

for i = 1:80

filename = ([`.jnartx(new)’ num2str(i)`.img’]);

nii = load_nii(filename);

x = nii.img(:,:,:);

t = x(BA 17);

Temp_array = [Temp_array t];

end

(The specified MATLAB program is to extract the data for Brodmann area 17).

Data for Brodmann areas 18 and 19 are extracted using similar code.

## Abbreviations

ANOVA: analysis of variance (statistical technique)
BA: Brodmann area (brain cortical areas)
EPI: echo planar imaging
fMRI: functional magnetic resonance imaging (technique to scan images)
FOV: field of view
GLM: generalized linear model (a statistical technique)
HCC: hallucination circle (visual stimulus)
HCF: hallucination fan (visual stimulus)
KAIST: Korea Advanced Institute of Science and Technology
LSD: least significant difference (statistical technique)
MLR: multiple linear regression
MNI: Montreal Neurological Institute
MPRAGE: magnetization-prepared rapid gradient-echo
MSE: mean square error
MT: middle temporal (visual area)
RTC: retinotopy circle (visual stimulus)
RTX: retinotopy cross (visual stimulus)
SPM: statistical parametric mapping
TE: echo time
TR: repetition time
V1: visual area 1
V2: visual area 2
V3: visual area 3
V4: visual area 4
V5: visual area 5
V6: visual area 6
